# Stereoselective Reduction of Imines with Trichlorosilane Using Solid-Supported Chiral Picolinamides

**DOI:** 10.3390/molecules21091182

**Published:** 2016-09-06

**Authors:** Sílvia D. Fernandes, Riccardo Porta, Pedro C. Barrulas, Alessandra Puglisi, Anthony J. Burke, Maurizio Benaglia

**Affiliations:** 1 Department of Chemistry and Chemistry Center of Évora, School of Science and Technology and Institute for Research and Advanced Studies, University of Évora, Rua Romão Ramalho 59, Évora 7000, Portugal; silvia.fernandes@gmail.com (S.D.F.); pedro.barrulas@gmail.com (P.C.B.); 2 Dipartimento di Chimica, Università degli Studi di Milano, Via Golgi 19, Milano 20133, Italy; alessandra.puglisi@unimi.it

**Keywords:** supported catalysis, catalytic reactors, trichlorosilane, chiral picolinamides, organocatalysis, flow chemistry

## Abstract

The stereoselective reduction of imines with trichlorosilane catalyzed by chiral Lewis bases is a well-established procedure for the synthesis of enantio-enriched amines. Five supported cinchona-based picolinamides have been prepared and their activity tested in a model reaction. The comparison of different supporting materials revealed that polystyrene gave better results than silica in terms of stereoselectivity. The applicability of the solid-supported catalyst of choice to the reduction of different imines was also demonstrated. Additionally, for the first time, a catalytic reactor containing a polymer-immobilized chiral picolinamide has been employed for the stereoselective reduction of imines with trichlorosilane under continuous flow conditions.

## 1. Introduction

Nowadays, chiral organocatalysts can be successfully employed as alternative catalytic species to enzymes or chiral organometallic complexes to perform stereoselective transformations [1]. However, the main issue associated with the use of organic molecules as catalysts is the need of high loadings (typically 1–10 mol %) when compared to other systems. The immobilization of the catalysts onto a solid support can overcome this drawback favoring an easy recovery and recycling of the catalytic species [2,3,4]. More recently, the combination of supported catalysts with continuous flow processes resulted in the development of catalytic reactors: [5,6] in these systems, the insoluble catalyst is confined to the reactors and purification steps necessary to isolate the product are significantly reduced [7]. Due to the absence of coordinated metal species, solid supported organocatalysts are particularly suitable to the application in catalytic reactors, since the risk of metal leaching is intrinsically avoided [8,9].

Among the metal-free methodologies available for the enantioselective reduction of imines [10,11], the use of trichlorosilane represents one of the most successful ways. The low cost of the reducing agent made the reduction method very attractive and pushed numerous groups to develop several different classes of chiral Lewis bases able to guarantee excellent levels of enantioselectivity in the reduction of differently functionalized ketoimines [12,13,14,15,16].

In the literature, only a few reports on the stereoselective reduction of imines with trichlorosilane promoted by solid supported chiral organocatalysts have appeared. Kocovsky’s research group reported the use of supported chiral formamides derived from *N*-methyl valine for the reduction of ketoimines with HSiCl_3_. These catalysts were supported onto insoluble [17] and soluble polymers [18] as well as onto soluble dendrons [19]. These systems showed good reactivity, enantioselectivities up to 91%, and could be easily recycled. However, the application of supported chiral organocatalysts in a catalytic reactor for the reduction of imines with HSiCl_3_ is still unknown. Here we wish to report the first example of a continuous flow process for the stereoselective synthesis of chiral amines mediated by trichlorosilane using solid supported picolinamides.

## 2. Results and Discussion

### 2.1. Catalyst Synthesis

**Figure 1:**
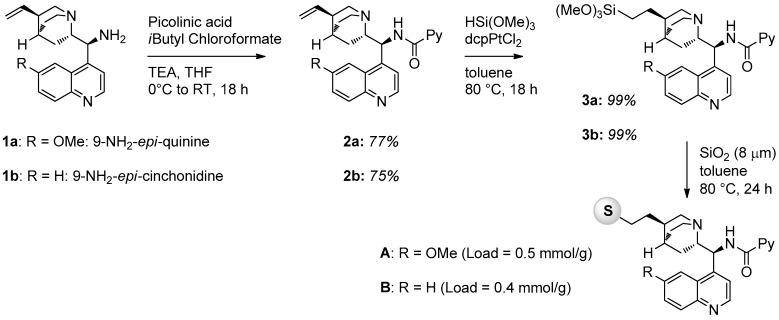
Synthesis of silica-supported catalysts **A** and **B**.

In this work, we prepared five solid supported chiral picolinamides derived from cinchona alkaloid primary amines using silica and polystyrene as supporting material. The general strategy to prepare silica supported catalysts involved the functionalization of the double bond of the quinuclidine ring with a trimethoxysilyl derivative, necessary to graft onto commercially available silica nanoparticles. The synthesis of catalysts **A** and **B** is illustrated in Figure 1. The primary amine derived from cinchona alkaloids (**1a** or **1b**) was converted into the corresponding picolinamide by reaction with picolinic acid and isobutyl chloroformate in the presence of triethylamine (TEA) in THF. The double bonds of compounds **2a** and **2b** were then quantitatively converted into trimethoxysilyl derivatives **3** by reaction with trimethoxysilane HSi(OMe_3_) using dcpPtCl_2_ as a catalyst for the hydrosilylation reaction. Final grafting was performed in the presence of commercially available SiO_2_ nanoparticles (Apex Prepsil Silica Media 8 μm) in toluene at 80 °C for 24 h. Catalyst loadings were determined by weight difference.

**Figure 2:**
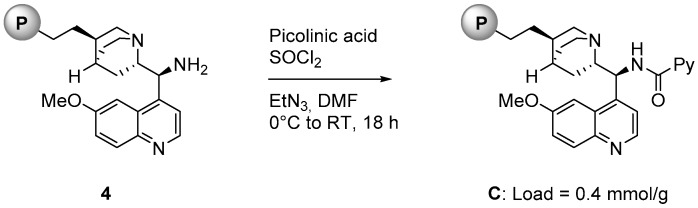
Synthesis of polystyrene-supported catalyst **C**.

As illustrated in Figure 2, polystyrene supported catalyst **C** was prepared starting from compound **4**, a supported organocatalyst available in our laboratories [20]. **4** was reacted with picolinic acid and thionyl chloride in the presence of triethylamine in DMF for 18 h at room temperature. The loading of **C** (0.4 mmol/g) was determined from the loading of the starting material **3** [21].

**Figure 3:**
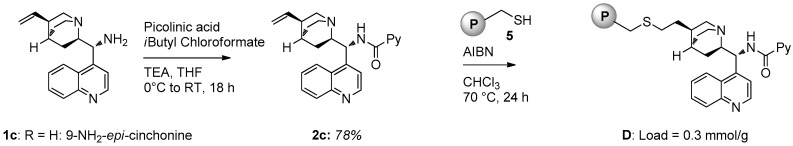
Synthesis of polystyrene-supported catalyst **D**.

For the preparation of polystyrene supported catalyst **D** an alternative strategy was employed (Figure 3): chiral picolinamide **2c** derived from 9-amino-epi-cinchonine was subjected to radical initiated thiol-ene coupling (initiated by AIBN) with solid **5**, a polystyrene bearing a thiol functionality [22]. The loading of **D** (0.3 mmol/g) was determined by weight difference.

**Figure 4:**
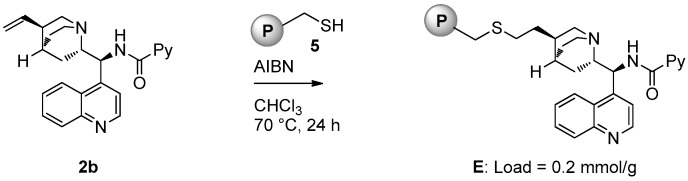
Synthesis of polystyrene-supported catalyst **E**.

Catalyst **E** (loading = 0.2 mmol/g) was prepared starting from picolinamide **2b** following the same strategy used for the preparation of **D** (Figure 4).

### 2.2. Stereoselective Reduction of Imines with HSiCl_3_ in Batch

The solid-supported catalysts **A**–**E** were tested in the reduction of imine **6a** with HSiCl_3_ in CH_2_Cl_2_ at room temperature for 18 h. Silica supported catalyst **A** gave the corresponding amine **7a** in moderate yield and low ee (Table 1, entry 1). The use of catalyst **B** resulted in a complete conversion of the starting material and the enantioselectivity improved up to 62% (entry 2). The use of polystyrene-supported catalyst **C** afforded chiral amine **7a** in 95% yield but very low ee of 23% (entry 3); this was probably due to an incomplete conversion of primary amine **4** into **C** [23]. Gratifyingly, upon using polystyrene supported catalyst **D**, the reaction proceeded in quantitative yield and 90% ee (entry 4), a result comparable to the one obtained with the homogeneous catalyst [24]. Lowering the catalyst loading to 10 mol % did not alter the reaction outcome (entry 5), while using 5 mol % of **D** resulted in slightly lower ee (entry 6). Using the pseudo enantiomer catalyst **E** amine **7a** obtained a 98% yield and a lower ee of 63% (entry 7).

Having established the best reaction conditions, we tested catalyst of choice **D** in the reduction of different substituted imines with HSiCl_3_ (Table 2). Electron poor imine **6b** was converted into the corresponding chiral amine **7b** with 93% yield and 85% ee (entry1). 4-Fluoro-substituted imine **6c** and 4-trifluoromethyl-substituted imine **6d** afforded the corresponding products **7c** and **7d** in quantitative yield and 87% and 84% ee, respectively (entries 2 and 3). The reaction with electron rich imine **6e** afforded amine **7e** in 96% ee and 85% ee (entry 4).

### 2.3. Continuous Flow Reaction

We next decided to focus our attention on the preparation of catalytic reactors in order to perform the stereoselective reduction of imines with trichlorosilane under continuous flow conditions. Preliminary studies showed that recovery and recycling of the immobilized catalyst were feasible: in the same conditions of Table 1, entry 4, polystyrene-supported picolinamide **D** was recycled three times. However, while after the first recycle (second run) the reaction promoted a 90% yield and 88% ee, already after the second recycle a clear decrease in enantioselectivity was observed (80% yield, 67% ee), that further dropped in the fourth run (77% yield, 37% ee). However, the behavior of the supported catalyst under continuous flow conditions was investigated.

A Omnifit glass column (i.d. = 1 cm, l = 10 cm) was filled with catalyst **D** (170 mg, 0.05 mmol) and was equipped with one fixed-length endpiece and one adjustable-length endpiece (bed length = 1 cm, internal volume = 0.78 mL): See Supplementary Materials. A 0.05 M solution of imine **6b** and trichlorosilane (5 equiv.) in dry dichloromethane was injected to the reactor through a syringe pump with a flow rate of 0.02 mL/min (residence time 25 min, determined experimentally) at room temperature. The outcome of the reactor was collected into a flask containing NaOH 10% solution. After phase separation and concentration, the product was isolated with no need for further purifications. Every 25 min, the product was collected and analyzed in order to determine the conversion and the ee. Results of the continuous flow experiments are reported in Table 3.

As reported in Table 3, the continuous flow process was run for 3 h and chiral amine **7b** was continuously obtained in very high yield. Unfortunately, the enantioselectivity of the process was quite low and it dropped to 47% ee (Table 3, entry 1) and to 20% after 150 h of operation (entry 7). Some organic residues coming from the catalyst degradation have been detected in the crude reaction mixture and that may account for the low enantioselectivity observed.

Overall, these results demonstrate how the use of a catalytic reactor can be advantageous in terms of product isolation and unit operations, however the system requires further studies in order to increase the stereoselectivity of the process.

## 3. Materials and Methods

### 3.1. General Information

Dry solvents were purchased and stored under nitrogen over molecular sieves (bottles with crown caps). Reactions were monitored by analytical thin-layer chromatography (TLC) using silica gel 60 F 254 pre-coated glass plates (0.25 mm thickness) and visualized using UV light. Flash chromatography was carried out on silica gel (230–400 mesh). Proton NMR spectra were recorded on spectrometers operating at 300 MHz (Bruker Fourier 300 or AMX 300, Milano, Italy). Proton chemical shifts are reported in ppm (δ) with the solvent reference relative to tetramethylsilane (TMS) employed as the internal standard (CDCl_3_ δ = 7.26 ppm). Commercial grade reagents and solvents were used without further purifications. Commercially available HSiCl_3_was freshly distilled under nitrogen atmosphere before use. Reagents mixtures were fed to continuous flow reactors using Syringe Pump Chemix Fusion 100. Continuous flow catalytic reactors were prepared using Glass Omnifit columns (Sigma-Aldrich, Milano, Italy) equipped with one adjustable-length end piece.

### 3.2. Catalysts Synthesis

Compound **2a**: Picolinic acid (1.5 mmol) was placed into a two necked flask and dissolved in dry THF (12 mL) under nitrogen atmosphere. Triethylamine (2.2 mmol) was added and the mixture was cooled to 0 °C. Ethyl chloroformate (1.5 mmol) was added dropwise and the mixture was stirred for 15 min at the same temperature, then 2 h at room temperature. After reaction time, a solution of **1a** [25] (1.5 mmol) in dry THF (8 mL), was added slowly. The reaction was stirred at room temperature for 3 h. After reaction time, H_2_O (3 mL) was added and the crude was concentrated in vacuum, diluted with AcOEt and then washed with H_2_O. Collected organic phases were dried with Na_2_SO_4_, concentrated in a vacuum, and the residue was purified by column chromatography on silica gel eluting with CH_2_Cl_2_:MeOH 95:5. Compound **2a** was obtained as brownish oil (1.04 mmol, 77% yield). All analytical data are in agreement with literature [24]. ^1^H-NMR (300 MHz, CDCl_3_) δ_H_ 8.88 (br s, 1H), 8.70 (d, *J* = 4 Hz, 1H), 8.46 (d, *J* = 4 Hz, 1H), 8.03–7.96 (m, 2H), 7.75 (d, *J* = 3 Hz, 1H), 7.71–7.66 (m, 1H), 7.42 (d, *J* = 4 Hz, 1H), 7.34–7.26 (m, 2H), 5.80–5.69 (m, 1H), 5.62 (br s, 1H), 5.01–4.91 (m, 2H), 3.94 (s, 3H), 3.44–3.36 (m, 1H), 3.33–3.23 (m, 2H), 2.81–2.68 (m, 2H), 2.27 (br, 1H), 1.66–1.48 (m, 4H), 0.96–0.88 (m, 1H).

Compound **2b**: prepared starting from **1b** [25] according to the procedure described for **2a** (1.25 mmol, 75% yield). ^1^H-NMR (300 MHz, CDCl_3_) δ_H_ 8.98 (br s, 1H), 8.89 (d, *J* = 4 Hz, 1H), 8.48–8.43 (m, 2H), 8.13 (d, *J* = 7 Hz, 1H), 8.03 (d, *J* = 2 Hz, 1H), 7.75–7.69 (m, 1H), 7.65–7.60 (m, 1H), 7.50–7.49 (d, *J* = 4 Hz, 1H), 7.37 (dd, *J* = 2 Hz, 5 Hz, 1H), 5.80–5.69 (m, 1H), 5.64 (br s, 1H), 5.03–4.94 (m, 2H), 3.42–3.18 (m, 3H), 2.88–2.73 (m, 2H), 2.31 (br s, 1H), 1.71–1.53 (m, 3H), 1.50–1.42 (m, 1H), 0.99–0.92 (m, 2H).

Compound **2c**: prepared starting from **1c** [25] according to the procedure described for **2a** (1.17 mmol, 78% yield). ^1^H-NMR (300 MHz, CDCl_3_) δ_H_ 9.04–8.93 (m, 1H), 8.85 (d, *J* = 4 Hz, 1H), 8.56 (d, *J* = 5 Hz, 1H), 8.46–8.40 (m, 1H), 8.11 (d, *J* = 8 Hz, 1H), 8.02–7.99 (m, 1H), 7.76–7.68 (m, 2H), 7.62–7.57 (m, 1H), 7.50–7.46 (m, 1H), 7.37–7.33 (m, 1H), 5.95–5.84 (m, 1H), 5.60 (br s, 1H), 5.15–5.07 (m, 2H), 3.28–3.19 (m, 1H), 3.11–2.91 (m, 4H), 2.33–2.22 (m, 1H), 1.67–1.61 (m, 1H), 1.57–1.45 (m, 2H), 1.35–1.24 (m, 1H), 1.04–0.93 (m, 1H).

Compound **3a**: **2a** (0.5 mmol) and (dcp)PtCl_2_ (0.025 mmol) were dissolved in dry THF (8 mL) under nitrogen atmosphere and trimethoxysilane HSi(OMe)_3_ (1.5 mmol) was slowly added. The mixture was stirred at 60 °C for 24 h. After reaction time, the solvent was evaporated under vacuum and **3a** was obtained as brownish oil (0.5 mmol, 99% yield). It was used in the following step without any further purification (the product easily degrades during column chromatography). ^1^H-NMR (300 MHz, CDCl_3_) δ_H_ 8.94 (bs, 1H), 8.71 (d, 1H), 8.52 (bs, 1H), 7.97–8.05 (m, 2H), 7.74 (bs, 2H), 7.43 (s, 1H), 7.33 (d, 2H), 6.61 (bs, 1H), 3.96 (s, 3H), 3.59 (s, 9H), 3.26–3.34 (m, 2H), 3.06 (bs, 1H), 2.80 (bs, 1H), 2.48 (bs, 1H), 1.86 (m, 2H), 1.59–1.72 (m, 3H), 1.35 (m, 2H), 0.98 (bs, 1H), 0.52 (m, 2H) ppm.

Compound **3b**: prepared starting from **2b** according to the procedure described for **3a** (0.5 mmol, 99% yield). It was used in the following step without any further purification (the product easily degrades during column chromatography). ^1^H-NMR (300 MHz, CDCl_3_) δ_H_ 9.28 (m, 1H), 8.91 (d, 1H), 8.49–8.54 (m, 2H), 8.13 (d, 1H), 8.06 (d, 1H), 7.63–7.76 (m, 4H), 7.36 (m, 1H), 5.97 (bs, 1H), 3.55 (s, 9H), 3.26–3.31 (m, 2H), 3.08 (bs, 1H), 2.80 (bs, 1H), 2.45 (bs, 1H), 1.88 (m, 2H), 1.54–1.72 (m, 3H), 1.33 (m, 2H), 1.09 (bs, 1H), 0.85 (m, 2H) ppm.

Catalyst **A**: Compound **3a** (0.5 mmol) was dissolved in dry toluene (10 mL) under a nitrogen atmosphere and then Apex Prepsil Silica Media 8 μm (0.5 g) was added. The mixture was stirred at 80 °C for 48 h after which it was then filtered. The solid was washed with dichloromethane (10 mL) and methanol (10 mL), and it was recovered and dried under high vacuum for 3 h. The organic layer was concentrated under a vacuum and unreacted compound **3a** was recovered. **A** was obtained as a yellowish solid; the loading was determined by weight difference (0.52 g, 0.5 mmol/g).

Catalyst **B**: prepared starting from **3b** according to the procedure described for **A** (0.52 g, 0.4 mmol/g).

Catalyst **C**: a mixture of **4** [20] (0.25 g, 0.1 mmol) and triethylamine (0.5 mmol) in dry THF (6 mL) was cooled to 0 °C. Then a solution of picolynoil chloride (0.3 mmol) in dry THF (3 mL) was slowly added under nitrogen atmosphere. The mixture warmed to rt and then stirred at 60 °C for 24 h. After reaction time the solid was filtered, washed with dichloromethane (10 mL) and methanol (10 mL), and it was recovered and dried under a high vacuum for 3 h. The loading of **C** was determined from the loading of the starting material (0.26 g, 0.4 mmol/g).

Catalyst **D**: **5** [22] (0.63 g, 1.0 mmol/g) was suspended in dry toluene (10 mL) under nitrogen atmosphere and then a solution of **2c** (1.0 mmol, g) and AIBN (1.0 mmol) in dry chloroform (3 mL) was slowly added. The mixture was stirred at 80 °C for 24 h under nitrogen atmosphere. After this reaction time, the solid was filtered and washed with CH_2_Cl_2_ (10 mL) and methanol (10 mL) and then dried under high vacuum for 3 h. The organic layer was concentrated in vacuum and unreacted compound **2c** was recovered and purified by column chromatography. The loading of **D** was determined by weight difference (0.65 g, 0.3 mmol/g). So far, solid state NMR spectroscopy did not give well defined spectra: further studies are underway to characterize the immobilized catalysts, also through solid-state IR spectroscopy and electron microscopy (SEM, TEM).

Catalyst **E**: prepared starting from **2b** and **5** according to the procedure described for **D** (0.65 g, 0.2 mmol/g).

### 3.3. Stereoselective Reduction of Imines

*General procedure for batch reaction*: supported catalyst (0.02 mmol) and imine (0.1 mmol) were introduced into a vial and dissolved in dry CH_2_Cl_2_ (1 mL) under inert atmosphere. HSiCl_3_ (1 M solution in CH_2_Cl_2_, 5 equiv.) was added at 0 °C and then the reaction was stirred at room temperature for 18 h. After this reaction time, the supported catalyst was removed by filtration and washed with dichloromethane. The organic layer was treated with NaOH 10% aq. until basic pH = 9. The organic layer was collected, dried with Na_2_SO_4_, and concentrated under a vacuum. The residue was purified by column chromatography on silica gel. The enantiomeric excess was determined by HPLC on chiral stationary phase.

*General procedure for continuous flow reaction:* a 0.05 M mixture of imine **6b** and HSiCl_3_ (5 equiv.) in dry CH_2_Cl_2_ was charged into a 2.5 mL SGE gas tight syringe and fixed on a syringe pump. The syringe was connected to the packed bed reactor and flushed at 0.02 mL/min. The reactor was then washed with pure CH_2_Cl_2_ at the same flow rate. Reactor outcome was collected into a flask containing CH_2_Cl_2_ and NaOH 10%. The outcome of the reactor was collected into a flask containing NaOH 10% solution. After phase separation and concentration, the product was isolated. Each 25 min the product was collected and analyzed. The yield was determined by NMR and the ee was determined on HPLC on chiral stationary phase.

### 3.4. Product Characterization

*(R)-4-Methoxy-N-(1-phenylethyl)aniline* **7a**: prepared according to the general procedure. The crude mixture was purified by column chromatography on silica gel eluting with hexane/ethyl acetate 95:5. **7a** was obtained as a yellowish solid. All analytical data are in agreement with literature. ^1^H-NMR (300 MHz, CDCl_3_) δ_H_ 7.43–7.26 (m, 5H), 6.73 (d, 2H), 6.58 (d, 2H), 4.46 (q, 1H), 3.74 (s, 3H), 1.58 (d, 3H) ppm. The enantiomeric excess was determined by HPLC on chiral stationary phase with Daicel Chiralcel OD-H column: eluent Hexane/iPrOH = 99/1, flow rate 0.8 mL/min, λ = 254 nm, τ_major_ = 22.3 min, τ_minor_ = 25.1 min.

*(R)-4-Methoxy-N-(1-(4-nitrophenyl)ethyl)aniline* **7b**: prepared according to the general procedure. The crude mixture was purified by column chromatography on silica gel eluting with hexane/ethyl acetate 95:5. **7b** was obtained as a yellowish solid. All analytical data are in agreement with the literature. ^1^H-NMR (300 MHz, CDCl_3_) δ_H_ 8.16–8.19 (m, 2H), 7.53–7.56 (d, 2H), 6.68–6.71 (m, 2H), 6.39–6.42 (m, 2H), 4.47–4.52 (q, 1H), 3.86 (bs, 1H), 3.69 (s, 3H), 1.52–1.53 (d, 3H) ppm. The enantiomeric excess was determined by HPLC on a chiral stationary phase with Daicel Chiralcel OD-H column: eluent Hexane/iPrOH = 8/2, flow rate 0.8 mL/min, λ = 254 nm, τ_major_ = 31.6 min, τ_minor_ = 37.7 min.

*(R)-N-(1-(4-Fluorophenyl)ethyl)-4-methoxyaniline* **7c**: prepared according to the general procedure. The crude mixture was purified by column chromatography on silica gel eluting with hexane/ethyl acetate 95:5. **7c** was obtained as a yellowish solid. All analytical data are in agreement with the literature. ^1^H-NMR (300 MHz, CDCl_3_) δ_H_ 7.28–7.33 (m, 2H), 6.95–7.00 (m, 2H), 6.68 (d, 2H), 6.43 (d, 2H), 4.37 (q, 1H), 3.68 (s, 4H), 1.45 (d, 3H) ppm. The enantiomeric excess was determined by HPLC on chiral stationary phase with Daicel Chiralcel OD-H column: eluent Hexane/iPrOH = 97/3, flow rate 1.0 mL/min, λ = 254 nm, τ_major_ = 14.2 min, τ_minor_ = 16.5 min.

*(R)-4-Methoxy-N-(1-(4-(trifluoromethyl)phenyl)ethyl)aniline* **7d**: prepared according to the general procedure. The crude mixture was purified by column chromatography on silica gel eluting with hexane/ethyl acetate 95:5. **7d** was obtained as a yellowish solid. All analytical data are in agreement with literature. ^1^H-NMR (300 MHz, CDCl_3_) δ_H_ 7.56 (d, 2H), 7.47 (d, 2H), 6.69 (d, 2H), 6.42 (d, 2H), 4.44 (q, 1H), 3.81 (bs, 1H), 3.68 (s, 3H), 1.48 (d, 3H) ppm. The enantiomeric excess was determined by HPLC on chiral stationary phase with Daicel Chiralcel OD-H column: eluent Hexane/iPrOH = 98/2, flow rate 0.8 mL/min, λ = 254 nm, τ_major_ = 17.7 min, τ_minor_ = 21.9 min.

*(R)-4-Methoxy-N-(1-(naphthalen-2-yl)ethyl)aniline* **7e**: Prepared according to the general procedure. The crude mixture was purified by column chromatography on silica gel eluting with hexane/ethyl acetate 95:5. **7e** was obtained as a yellowish solid. All analytical data are in agreement with literature. ^1^H-NMR (300 MHz, CDCl_3_) δ_H_ 7.78–7.82 (m, 4H), 7.41–7.51 (m, 3H), 6.65–6.69 (m, 2H), 6.48–6.52 (m, 2H), 4.53–4.58 (q, 1H), 3.88 (br, 1H), 3.66 (s, 3H), 1.55–1.57 (d, *J* = 6.8 Hz, 3H) ppm. The enantiomeric excess was determined by HPLC on chiral stationary phase with Daicel Chiralcel OD-H column: eluent Hexane/iPrOH = 98/2, flow rate 1.0 mL/min, λ = 254 nm, τ_major_ = 41.7 min, τ_minor_ = 52.7 min.

## 4. Conclusions

In conclusion, the immobilization of chiral Cinchona alkaloid-derived catalysts for the enantioselective, trichlorosilane-mediated reduction of ketoimines was investigated. A polystyrene-supported picolinamide was identified as a successful heterogeneous organocatalyst able to promote the C=N reduction of imines in high enantioselectivities, typically higher than 85% ee and up to 91% ee. Additionally, for the first time, a polymer-supported chiral organocatalyst was used in a catalytic reactor to perform the enantioselective reduction of imines in a continuous flow process. The flow systems showed promising results in terms of chemical activity, but lower enantioselectivities than those obtained in batch. Further studies aimed to improve the stereoselectivity of the process and the stability of the supported chiral catalyst are currently underway in our laboratories.

## Figures and Tables

**Table 1:**
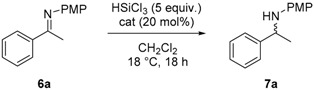
Screening of supported catalysts in the reduction of imine **6a** with HSiCl_3_.

Entry ^1^	Catalyst	Yield (%) ^2^	ee (%) ^3^
1	**A**	64	30 (*S*)
2	**B**	95	62 (*S*)
3	**C**	95	23 (*S*)
4	**D**	98	90 (*R*)
5 ^4^	**D**	95	91 (*R*)
6 ^5^	**D**	95	79 (*R*)
7	**E**	95	63 (*S*)

^1^ *Reaction conditions*: 0.1 mmol **6a**, 0.02 mmol supported catalyst, 0.5 mmol HSiCl_3_ in CH_2_Cl_2_ (1 mL); ^2^ isolated yield; ^3^ determined by HPLC on chiral stationary phase; ^4^ 10 mol % of **D** was used; ^5^ 5 mol % of **D** was used.

**Table 2:**
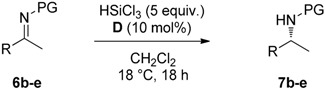
Reduction of various imines with HSiCl_3_ using polystyrene supported catalyst **D**.

Entry ^1^	R	PG	Yield (%) ^2^	ee (%) ^3^
1	**6b:** 4-NO_2_-C_6_H_4_-	PMP	93	85
2	**6c:** 4-F-C_6_H_4_-	PMP	98	87
3	**6d:** 4-CF_3_-C_6_H_4_-	PMP	97	84
4	**6e:** Naphtyl	Ph	96	85

^1^ *Reaction conditions*: 0.1 mmol **6**, 0.01 mmol **D**, 0.5 mmol HSiCl_3_ in CH_2_Cl_2_ (1 mL); ^2^ isolated yield; ^3^ determined by HPLC on chiral stationary phase.

**Table 3:**
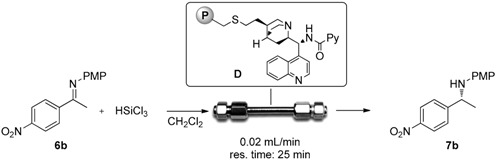
Continuous flow stereoselective reduction of imine **6b** with HSiCl_3_.

Entry	Running Time (min)	Yield (%) ^1^	ee (%) ^2^
1	0–50	98	47
3	50–75	98	44
4	75–100	93	30
5	100–125	93	25
6	125–150	87	22
7	150–175	86	20

^1^ Determined by ^1^H-NMR; ^2^ determined by HPLC on chiral stationary phase.

## Supplementary Materials

Supplementary materials can be accessed at: http://www.mdpi.com/1420-3049/21/9/1182/s1.

## References

[1] Okhuma T., Noyori R., Jacobsen E.N., Pfaltz A., Yamamoto H. (2004). Comprehensive Asymmetric Catalysis.

[2] Benaglia M. (2009). Recoverable and Recyclable Catalysts.

[3] Trindade A.F., Gois P.M.P., Afonso C.A.M. (2009). Recyclable Stereoselective Catalysts. Chem. Rev..

[4] Haraguchi N, Itsuno S. (2011). Polymeric Chiral Catalyst Design and Chiral Polymer Synthesis.

[5] Puglisi A., Benaglia M., Chiroli V. (2013). Stereoselective organic reactions promoted by immobilized chiral catalysts in continuous flow systems. Green Chem..

[6] Tsubogo T., Ishiwata T., Kobayashi S. (2013). Asymmetric Carbon-Carbon Bond Formation under Continuous-Flow Conditions with Chiral Heterogeneous Catalysts. Angew. Chem. Int. Ed..

[7] Porta R., Benaglia M., Puglisi A. (2016). Flow Chemistry: Recent Developments in the Synthesis of Pharmaceutical Products. Org. Proc. Res. Dev..

[8] Cantillo D., Kappe C.O. (2014). Immobilized Transition Metals as Catalysts for Cross-Couplings in Continuous Flow—A Critical Assessment of the Reaction Mechanism and Metal Leaching. ChemCatChem.

[9] Gursel I.V., Noel T., Wang Q., Hessel V. (2015). Separation/recycling methods for homogeneous transition metal catalysts in continuous flow. Green Chem..

[10] Benaglia M., Genoni A., Bonsignore M., Torres R.R. (2012). Enantioselective organocatalytic reductions. Stereoselective Organocatalysis: From C-C to C-Heteroatom Bond Formation.

[11] Rossi S., Benaglia M., Massolo E., Raimondi L. (2014). Organocatalytic strategies for enantioselective metal-free reductions. Catal. Sci. Technol..

[12] Guizzetti S., Benaglia M. (2010). Trichlorosilane-Mediated Stereoselective Reduction of C=N Bonds. Eur. J. Org. Chem..

[13] Jones S., Warner C.J.A. (2012). Trichlorosilane mediated asymmetric reductions of the C=N double bon. Org Biomol. Chem..

[14] Malkov A.V., Mariani A., MacDougall K.N., Kocovsy P. (2004). Role of Noncovalent Interactions in the Enantioselective Reduction of Aromatic Ketimines with Trichlorosilane. Org. Lett..

[15] Ye J., Wang C., Chen L., Wu X., Zhou L., Sun J. (2016). Chiral Lewis Base-Catalyzed, Enantioselective Reduction of Unprotected β-Enamino Esters with Trichlorosilane. Adv. Synth. Catal..

[16] Wang T., Di X., Wang C., Li Z., Sun J. (2016). Reductive Hydrazination with Trichlorosilane: A Method for the Preparation of 1,1-Disubstituted Hydrazines. Org. Lett..

[17] Malkov A.V., Figlus M., Kocovsy P. (2008). Polymer-Supported Organocatalysts: Asymmetric Reduction of Imines with Trichlorosilane Catalyzed by an Amino Acid-Derived Formamide anchored to a Polymer. J. Org. Chem..

[18] Malkov A., Figlus M., Prestly M.R., Rabani G., Cooke G., Kocovsky P. (2009). Soluble Polymer-Supported Organocatalysts: Asymmetric Reduction of Imines with Trichlorosilane Catalyzed by an Amino Acid Derived Formamide Anchored to a Soluble Polymer. Chem. Eur. J..

[19] Figlus M., Caldwell S.T., Walas D., Yesilba G., Cooke G., Kocovsy P., Malkov A.V., Sanyal A. (2010). Dendron-anchored organocatalysts: The asymmetric reduction of imines with trichlorosilane, catalysed by an amino acid-derived formamide appended to a Dendron. Org. Biomol. Chem..

[20] Porta R., Benaglia M., Coccia F., Cozzi F., Puglisi A. (2015). Solid Supported 9-Amino-9-deoxy-epi-quinine as Efficient Organocatalyst for Stereoselective Reactions in Batch and Under Continuous Flow Conditions. Adv. Synth. Catal..

[B21-molecules-21-01182] 21.Attempts to monitor the reaction from **3** to **C** via solid state NMR were unsuccessful; a complete conversion of the primary amine into the corresponding picolinamide could not be confirmed.

[22] Braslau R., Rivera F., Tansakul C. (2013). Reversible crosslinking of polymers bearing pendant or terminal thiol groups prepared by nitroxide-mediated radical polymerization. React. Funct. Polym..

[B23-molecules-21-01182] 23.The reaction in the presence of polystyrene supported primary amine **4** as catalyst gave amine **7a** in 45% yield as a racemic mixture.

[24] Barrulas P.C., Genoni A., Benaglia M., Burke A.J. (2014). Cinchona-Derived Picolinamides: Effective Organocatalysts for Stereoselective Imine Hydrosilylation. Eur. J. Org. Chem..

[25] Brunner H., Bugler J., Nuber B. (1995). Preparation of 9-amino-(9-deoxy) cinchona alkaloids. Tetrahedron Asymmetry.

