# Supplementary material for: Stereoselective Reduction of Imines with Trichlorosilane Using Solid-Supported Chiral Picolinamides
Source: Molecules. 2016 Sep 6;21(9):1182. doi: 10.3390/molecules21091182 (PMC6274114; doi:10.3390/molecules21091182)
Supplement: Supplementary file 1 [file molecules-21-01182-s001.pdf]

Supplementary Materials: Stereoselective Reduction of Imines with Trichlorosilane Using Solid-Supported Chiral Picolinamides

Sílvia D. Fernandes, Riccardo Porta, Pedro C. Barrulas, Alessandra Puglisi, Anthony J. Burke and Maurizio Benaglia

Set-up of continuous flow experiment

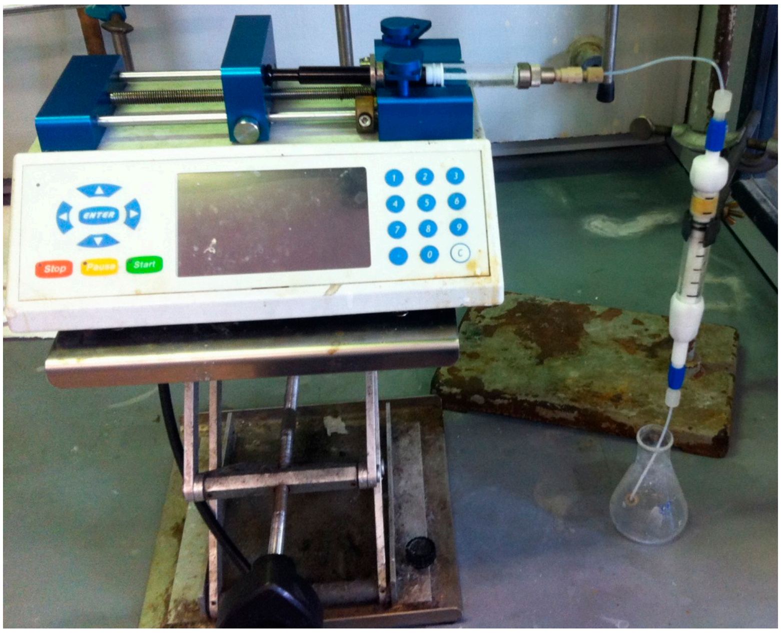

Figure S1. Set-up of continuous flow experiment;

HPLC traces

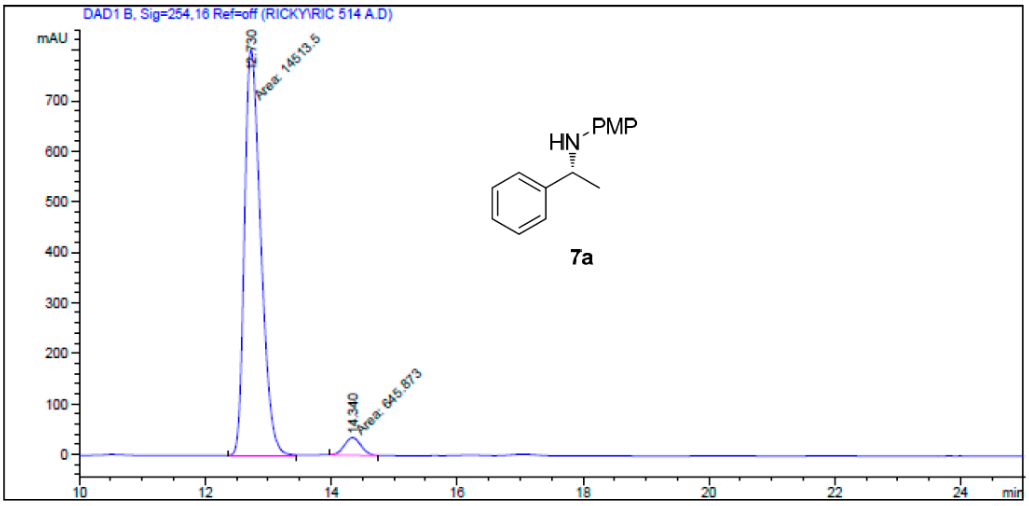


```
Signal 1: DAD1 B, Sig=254,16 Ref=off

Peak RetTime Type  Width     Area      Height     Area
  #   [min]        [min]   [mAU*s]     [mAU]        %
----|-------|----|-------|----------|----------|--------|
   1  12.730 MM    0.3015 1.45135e4  802.19348  95.7394
   2  14.340 MM    0.3101  645.87299   34.71146   4.2606

Totals :                  1.51593e4  836.90495
```

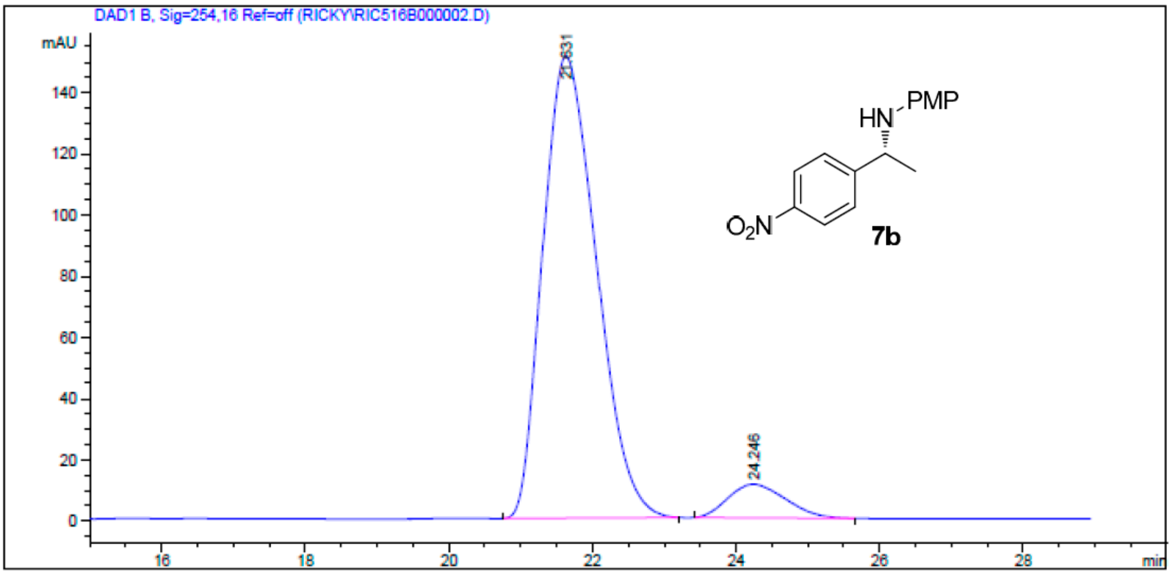

Signal 1: DAD1 B, Sig=254,16 Ref=off

Peak #	RetTime [min]	Type	Width [min]	Area [mAU*s]	Height [mAU]	Area %
1	21.631	BB	0.8416	7839.49072	151.02728	92.6847
2	24.246	BB	0.7331	618.74463	11.01985	7.3153
Totals :				8458.23535	162.04713	

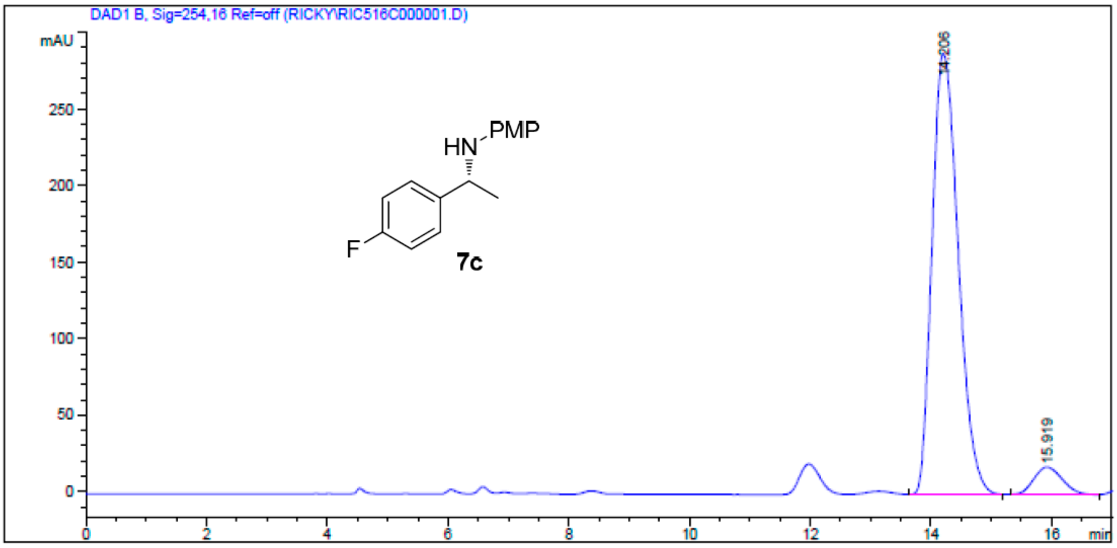

Peak #	RetTime [min]	Type	Width [min]	Area [mAU*s]	Height [mAU]	Area %
1	14.206	VB	0.4700	8699.45215	288.31537	93.4443
2	15.919	BB	0.5228	610.31989	18.21909	6.5557
Totals :				9309.77203	306.53446	

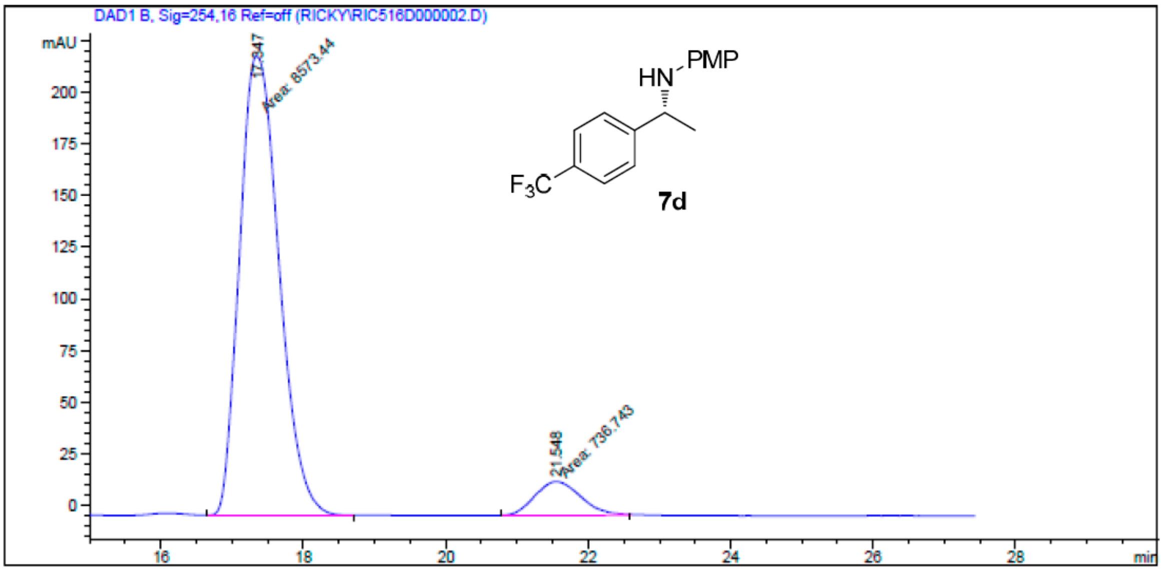

Peak #	RetTime [min]	Type	Width [min]	Area [mAU*s]	Height [mAU]	Area %
1	17.347	MM	0.6429	8573.43555	222.26537	92.0867
2	21.548	MM	0.7528	736.74268	16.31135	7.9133
Totals :				9310.17822	238.57672	

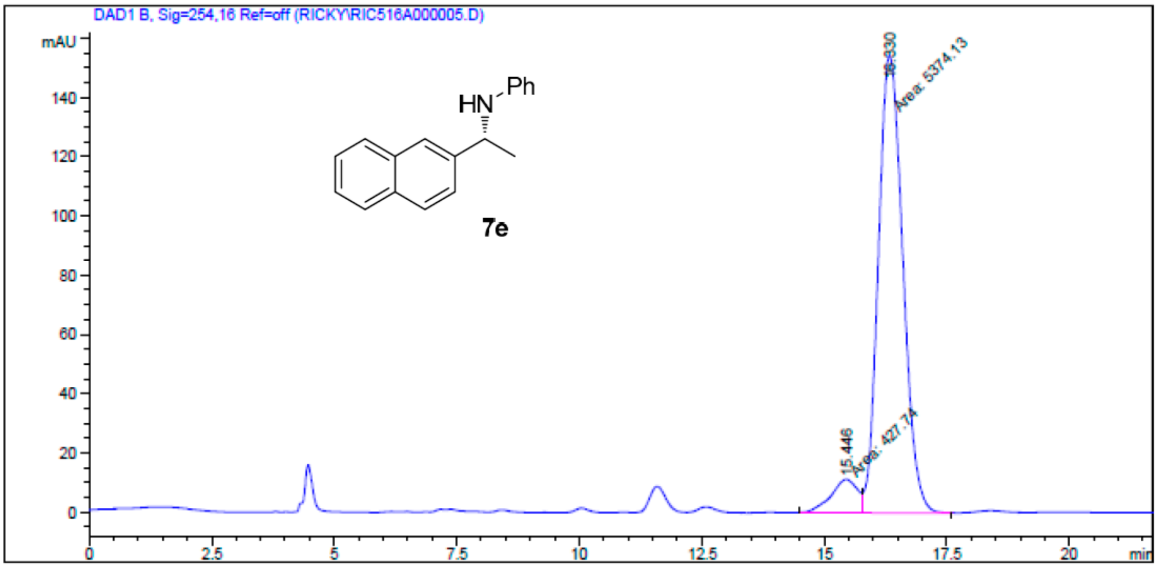

Peak #	RetTime [min]	Type	Width [min]	Area [mAU*s]	Height [mAU]	Area %
1	15.446	MF	0.6341	427.73965	11.24334	7.3724
2	16.330	FM	0.5798	5374.13086	154.48079	92.6276
Totals :				5801.87051	165.72413	
